# Development of an Artificial Intelligence System for the Automatic Evaluation of Cervical Vertebral Maturation Status

**DOI:** 10.3390/diagnostics11122200

**Published:** 2021-11-25

**Authors:** Jing Zhou, Hong Zhou, Lingling Pu, Yanzi Gao, Ziwei Tang, Yi Yang, Meng You, Zheng Yang, Wenli Lai, Hu Long

**Affiliations:** 1 State Key Laboratory of Oral Diseases, National Clinical Research Center for Oral Diseases, Department of Orthodontics, West China Hospital of Stomatology, Sichuan University, Chengdu 610041, China; nkuzhoujing@163.com (J.Z.); zh17736839697@163.com (H.Z.); dangdapll@163.com (L.P.); yanmei100@163.com (Y.G.); tziwei2016@163.com (Z.T.); yy18583233717@163.com (Y.Y.); wenlilai@scu.edu.cn (W.L.); 2 State Key Laboratory of Oral Diseases, National Clinical Research Center for Oral Diseases, Department of Oral Radiology, West China Hospital of Stomatology, Sichuan University, Chengdu 610041, China; youmeng@scu.edu.cn; 3 State Key Laboratory of Oral Diseases, National Clinical Research Center for Oral Diseases, Department of General Dentistry, West China Hospital of Stomatology, Sichuan University, Chengdu 610041, China; hxkqyx@163.com

**Keywords:** artificial intelligence, cervical vertebral maturation, skeletal age, deep learning, convolutional neural network, orthodontics

## Abstract

Background: Cervical vertebral maturation (CVM) is widely used to evaluate growth potential in the field of orthodontics. This study is aimed to develop an artificial intelligence (AI) system to automatically determine the CVM status and evaluate the AI performance. Methods: A total of 1080 cephalometric radiographs, with the age of patients ranging from 6 to 22 years old, were included in the dataset (980 in training dataset and 100 in testing dataset). Two reference points and thirteen anatomical points were labelled and the cervical vertebral maturation staging (CS) was assessed by human examiners as gold standard. A convolutional neural network (CNN) model was built to train on 980 images and to test on 100 images. Statistical analysis was conducted to detect labelling differences between AI and human examiners, AI performance was also evaluated. Results: The mean labelling error between human examiners was 0.48 ± 0.12 mm. The mean labelling error between AI and human examiners was 0.36 ± 0.09 mm. In general, the agreement between AI results and the gold standard was good, with the intraclass correlation coefficient (ICC) value being up to 98%. Moreover, the accuracy of CVM staging was 71%. In terms of F1 score, CS6 stage (85%) ranked the highest accuracy. Conclusions: In this study, AI showed a good agreement with human examiners, being a useful and reliable tool in assessing the cervical vertebral maturation.

## 1. Introduction

Dental malocclusion, with a prevalence of 20–83% among both adolescents and adults, manifests as misaligned teeth resulting in poor masticatory function and esthetic problems [1,2,3,4]. Of particular, among adolescents, early interventions could eliminate or intercept the development of malocclusion [5], e.g., mandibular advancement therapy for adolescents with mandibular retrusion [6]. To ensure the success of early interventions, meticulous and correct assessment of growth potential and the timing of growth spurt is very important. Traditionally, skeletal age was used to assess the growth potential, among which hand-wrist bone age and cervical vertebral maturation (CVM) staging were widely used by dental practitioners [7,8]. Since hand-wrist radiographs require additional radiographic examinations, both orthodontists and patients are reluctant to use this method in orthodontic practice. Fortunately, since CVM staging could be assessed in lateral cephalograms that are required for orthodontic diagnosis, CVM staging has been gaining more and more popularity among orthodontists. CVM is determined through the morphological changes of the bodies of the second, third and fourth cervical vertebrae (C2–C4) on lateral cephalograms [8]. However, it is difficult and time-consuming for practitioners to determine skeletal maturation and growth spurt through CVM staging correctly.

Nowadays, artificial intelligence (AI) has gained a giant leap in dentistry, assisting clinicians in a variety of fields, e.g., detection of periapical lesions and root fractures, optimizing implant designs, diagnosis of oral cancer [9,10,11]. Deep learning is a key field of AI, which uses a learning model to extract features of the labelled dataset and eventually can predict labels on a new dataset [12]. Mimicking the way that human brain neurons signal to another, neural networks are widely used in deep learning.

Over the past decade, several researchers have explored the promising application of AI in analysis of cephalometric images. Hwang et al. used a customized You-Only-Look-Once version 3 algorithm (YOLOv3) to detect 80 landmarks on 1028 cephalograms. The mean detection error between AI and human was up to 1.46 ± 2.97 mm [13]. Larson et al. used a deep residual network to estimate skeletal maturity on pediatric hand radiographs and reported a similar accuracy to an expert radiologist [14]. Moreover, Kok et al. and Amasya et al. pioneered the applications of neural networks in CVM assessment [15,16]. However, they included 300 and 647 images in the aforementioned two studies, respectively. Nevertheless, they didn’t list the distribution of the dataset. We inferred that there were 50 and 108 images on average for AI learning for each CVM stage. Considering the short duration of growth and development, the number of CS 3 which means the growth spurt was insufficient for AI to learn. Therefore, the generalization of their results is limited by small sample sizes (*n*= 300 and 647 for Kok et al. and Amasya et al., respectively) and only studying the agreement between AI and actual CVM staging.

Therefore, in this study, we developed an AI system to automatically assess CVM based on a larger sample size (*n* = 1080) and to assess other indices (e.g., sensitivity), in order to comprehensively evaluate the generalization potential of an AI system for CVM staging.

## 2. Materials and Methods

**Figure 1:**
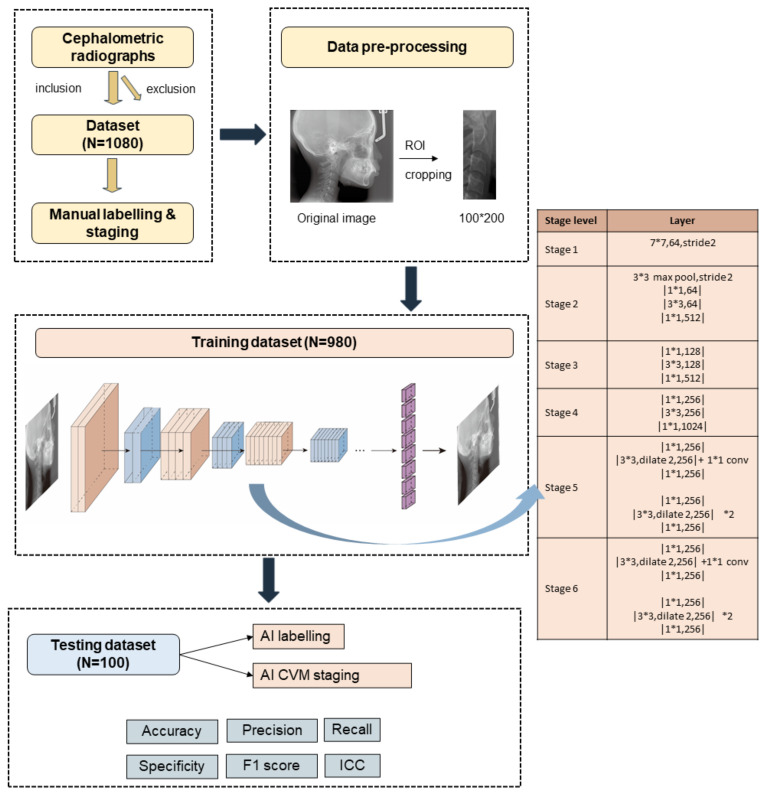
The experimental design of the study. Step 1: inclusion and exclusion. Step 2: data pre-processing. Step 3: model training and testing. Step 4: performance evaluation.

This study was approved by Ethical Committee of West China Hospital of Stomatology, Sichuan University. For better understanding, we reported this study according to a checklist prompted by Schwendicke et al. [17]. The experimental design of the study is summarized in Figure 1. Briefly, 1080 images were selected and labelled by human examiners. After pre-processing, they were divided into training and testing dataset. The training dataset of 980 labelled images was input to an AI-based system for machine learning. Finally, 100 images were used to test the AI system and statistical analysis was conducted to evaluate AI performance.

### 2.1. Patients and Dataset

Cephalometric radiographs of patients with a chronological age between 6 and 22 years old were obtained from Department of oral radiology, West China Hospital of Stomatology, Sichuan University. Only images with clearly identified contours of the second (C2), third (C3) and fourth (C4) cervical vertebrae were included. Patients with congenital diseases were excluded from the dataset. In case of several images from the same patient, we deleted the repeated ones based on their medical record number. Therefore, the images in the training set and the testing set were from different patients. Finally, 1080 images (jpg format) were included and randomly assigned into training and testing dataset (Table 1).

### 2.2. Manual CVM Staging

The CVM staging (CS) of 1080 images were identified by two examiners independently and in duplicate (J.Z. & H.Z., who had three years’ experience in CVM assessment) according to Baccetti methods [8]. Briefly, the CVM assessment was based on the morphology changes of C2, C3 and C4. At CS1, the lower border of C2, C3 and C4 were flat and presented no concavity. Moreover, the shape of C3 and C4 were trapezoid in shape. With growth and development, the concavity of lower border of C2, C3, C4 became more obvious and the height of C3 and C4 increased, therefore the shape of C3, C4 changed to horizontal rectangles, squares or vertical rectangles. Disagreements were resolved by a third examiner (H.L., who had 10 years’ relevant experience). The final CVM stages were served as a gold standard. The CS of both the training and testing datasets are displayed in Table 1. For inter-rater reliability, the kappa value was 0.86 and the intraclass correlation efficient (ICC) value was 0.98.

### 2.3. Manual Labelling

**Figure 2:**
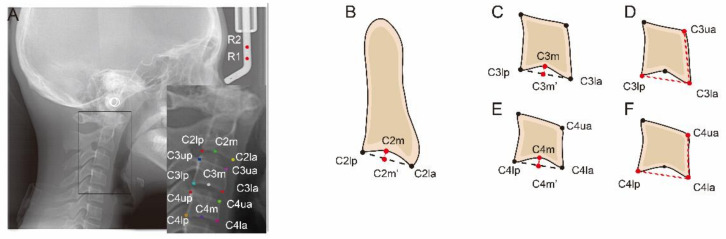
Schematic representation of anatomic landmarks and liner measurements for the determination of cervical vertebral morphology. (**A**) Two reference points (R1 and R2) and thirteen anatomic landmarks of C2, C3 and C4. The distance between the two reference points (R1 and R2) is 10 mm. (**B**) C2Conc = C2m − C2m′. (**C**) C3Conc = C3m − C3m′. (**D**) C3BAR = (C3lp − C3la)/(C3ua − C3la). (**E**) C4Conc = C4m − C4m′. (**F**) C4BAR = (C4lp − C4la)/(C4ua − C4la).

The training and testing dataset was uploaded to an open-source annotation tool named LabelMe (https://github.com/wkentaro/labelme; accessed on 18 February 2021). Then, as presented in Figure 2, two reference landmarks and thirteen anatomic landmarks were manually labelled on each cephalometric image by an examiner (J.Z.) in duplicate after a three-month interval. The distance between two reference points is 10 mm. These landmarks were used for linear and ratio measurements. The definitions of landmarks and measurements are shown in Table 2. Then the labelled films were saved as. json format and input to the model for training and testing.

### 2.4. Model Training and Testing

**Figure 3:**
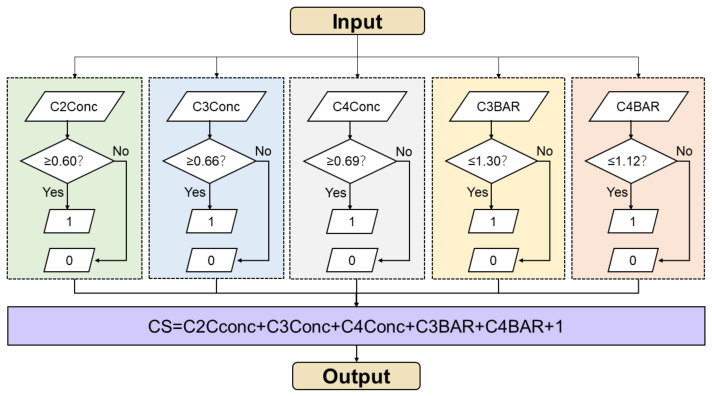
Workflow of the AI system to assess the CVM stage. Measurements are input and reassigned according to thresholds. The CVM stages were calculated and output.

To reduce interference from other anatomic structures, a final ROI (region of interest) included all part of C2–C4 was cropped on images with a size of 100* × 200 pixels (Figure 1). The preprocessing images were input to a convolutional neural network (CNN) for training and testing. The experiments were performed on Intel core i7 quadra core processor with Nvidia 1080 graphics card. The models were developed with the pytorch libraries using the Python programming language. We used a Detnet architecture with relu activation function, adam optimization and MSE loss function to conduct machine learning. Based on resnet50, Detnet introduced the extra stages in the backbone and was more powerful in locating large objects and finding small objects [18]. The model consisted of 58 convolution layers and 1 fully connected layer. In the training stage, we set the training epochs to 200 and batch size as 32. The learning rate was set as 0.0001 and epsilon was 1 × 10^−8^. After training on 980 radiographic images, the CNN model achieved satisfactory results for new images. To test AI performance, 100 new images were uploaded. Automatically, the CNN model labelled 2 reference landmarks and 13 anatomic landmarks on each image. These landmarks were output in fully connected layer. Afterwards, the linear and ratio measurements (C2conc, C3conc, C4conc, C3BAR, C4BAR) were calculated and the CVM stage was output according to a workflow shown in Figure 3. In brief, these measurements were input and estimated with thresholds from Baccetti’s original data [8].

### 2.5. Statistical Analysis

All the statistical analysis were performed using SPSS Statistics Version 22.0. The inter-rater reliability of the manual CVM staging was determined through kappa coefficient and intra-class correlation coefficient (ICC) test. The intra-rater reliability of the labelling work was calculated through mean difference and the mean value of labelling was served as gold standard. The disagreement between AI and manual labelling were calculated in terms of distances measured in millimeter scales. Moreover, both linear measurements (C2Conc, C3Conc, C4Conc) and ratio measurements (C3BAR, C4BAR) were performed. The differences of the aforementioned measurements between AI and manual performance were compared. As for CVM staging difference between AI and gold standard, the overall accuracy and ICC were calculated and the accuracy of each stage was calculated in precision, recall, specificity, F1 score [19]. The formulas of these metrics are listed as follows:

Accuracy = $\frac{TP+TN}{TP+TN+FN+FP}$, which is a general evaluation of AI performance.
Precision = $\frac{TP}{TP+FP}$ = positive predictive value (PPV), which presents the ability of AI to correctly predict positives.
Recall = $\frac{TP}{TP+FN}$ = sensitivity, which reflects ability of AI to find all the positive samples.
Specificity = $\frac{TN}{TP+FN}$, which reflects the ability of AI to find all the negative samples.
F1 score = $\frac{2*pecison*recall}{precision+recall}$, which weight precision and recall harmoniously to completely evaluate AI performance.

TP (true positive) is the number of AI correctly predicted images and FP (false positive) is the number of AI incorrectly predicted image while human classified as positive in a binary task. In similar, TN (true negative) and FN (false negative) are the number of AI correctly predicted images and incorrectly predicted image while human classified as negative, respectively.

## 3. Results

### 3.1. Evaluation of Labelling

**Figure 4:**
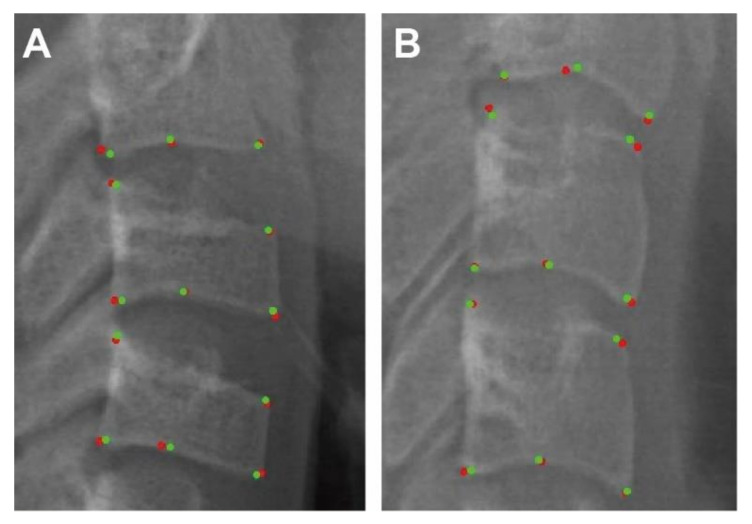
CNN output results: Anatomic landmarks that AI (points in red) and human (points in green) labelled in testing dataset. (**A**). AI and human labelled landmarks for CS 3 (**B**). AI and human labelled landmarks for CS6.

**Figure 5:**
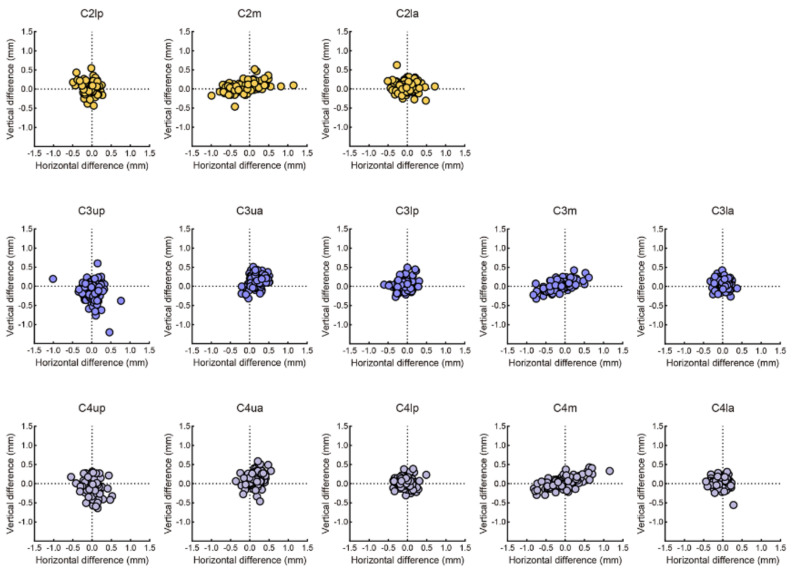
Scattergram representation of the labelling difference between AI and human. The mean differences between AI and human of C2 landmarks (line 1) were 0.29 ± 0.25 mm, 0.47 ± 0.33 mm, 0.35 ± 0.27 mm, respectively. The mean differences between AI and human of C3 landmarks (line 2) were 0.48 ± 0.38 mm, 0.42 ± 0.24 mm, 0.24 ± 0.15 mm, 0.37 ± 0.28 mm, 0.24 ± 0.17 mm, respectively. The mean differences between AI and human of C4 landmarks (line 3) were 0.42 ± 0.36 mm, 0.43 ± 0.27 mm, 0.25 ± 0.21 mm, 0.46 ± 0.27 mm, 0.27 ±± 0.18 mm, respectively.

The mean differences between the first and second manual labelling were 0.48 ± 0.12 mm. Moreover, the mean differences between the manual labelling (gold standard) and the AI labelling were 0.36 ± 0.09 mm. Two examples of the landmarks that AI (points in red) and human (points in green) has labelled were displayed in Figure 4. To visualize and evaluate the error pattern in two-dimensional space, scattergrams were depicted. As displayed in Figure 5, the AI and human labelling were well matched. The differences between the first and second manual labelling and between the manual and AI labelling for each point are detailed in Table 3.

### 3.2. Evaluation of Measurements

**Figure 6:**
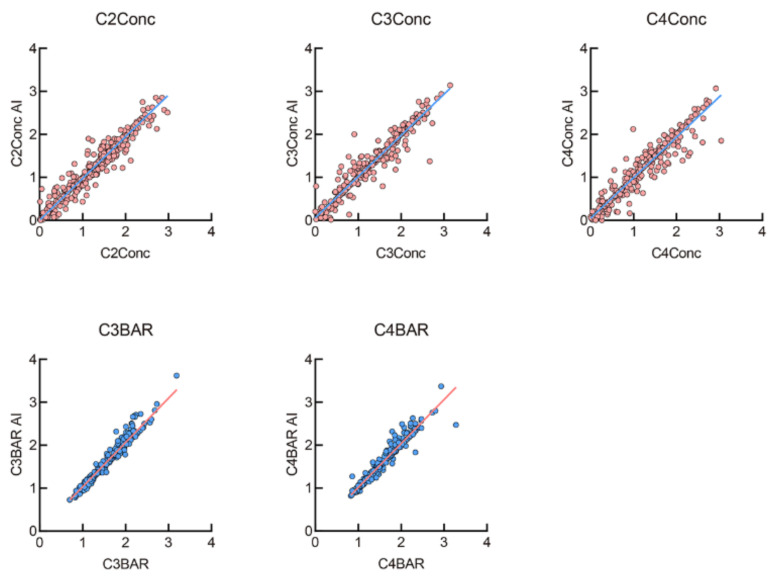
The linear and ratio measurements difference between AI and human. They were well-matched and fitted into Y = X, R^2^ = 0.93, 0.94, 0.93, 0.97 and 0.95, respectively; all *p* < 0.001).

The linear and ratio measurements were calculated according to manual- and AI-labelled anatomic points respectively. As displayed in Figure 6, the results revealed that they were well-matched and fitted into a linear function (Y = X) (R^2^ = 0.93, 0.94, 0.93, 0.97 and 0.95, respectively; all *p* < 0.001). Moreover, the ICC for all the five measurements were greater than 0.90, indicating that AI performed comparably with human examiners.

### 3.3. Evaluation of AI Staging

For manual CVM staging, our results revealed that the inter-rater reliability was 0.86, indicative of perfect agreement between the two examiners. With the manual CVM staging as the gold standard, the 71% general accuracy was observed for AI CVM staging. In terms of F1 score, CS6 ranked the highest accuracy (85%) and CS1 the second. CS3, which indicated the growth spurt, was the lowest (31%). The overall ICC value was 0.98, indicating that the manual and AI CVM staging was in perfect agreement. In particular, the precision, recall and specificity ranging from 25–100%,36–100% and 84–100% respectively. More details were listed in Table 4.

## 4. Discussion

The assessment of growth and development plays a critical role in orthodontic treatment planning for growing patients. Appropriate growth prediction and prudent treatment timing benefit adolescents with skeletal discrepancy through growth modification by utilizing growth potential. Several indicators have been used to determine the growth potential, i.e., chronological age, body height, sexual maturity, dental age and skeletal age. Great individual variations have been discovered in chronological age, increase in body height and sexual maturity [20,21]. As for dental age, dentition phase and dental maturity are often used to assess skeletal maturity. However, a large body of evidence indicated that dental age is not recommended to assess skeletal maturity or to determine growth spurt [22,23,24]. Due to high reliability, skeletal age is more popular in the dental community. Larson et al. applied CNN network in hand-wrist radiographs method and reported a good agreement. Compared with the hand-and-wrist method [25,26], CVM staging method based on cervical vertebrae is more popular among orthodontists for no additional radiographic requirements. Since the introduction of a modified CVM staging method by Baccetti et al. [8], this CVM staging has been consistently proved to be reliable [25,27,28].

The CVM stages are determined through the morphological changes of the contours of cervical vertebrae (C2–C4). With the aid of deep learning algorithm, CVM assessment can be more accurate and efficient. As pixel values could be digitally coded, radiology images are easily translated into computer language [29]. Several seminal studies have successfully translated AI applications into identifying cephalometric landmarks [30,31]. The first step of the AI CVM staging was to identify the contours of the cervical vertebrae. Our results revealed that the AI labelling was in almost perfect with the gold standard (manual labelling), with the mean error being 0.36 mm that was even smaller than that (0.48 mm) between the two manual labellings. This suggested that AI labelling was more consistent with manual labelling. It has been revealed that automatic identification of landmarks is considered to be successful if the difference between AI and human is less than 2 mm [32], indicating that the AI labelling in our present study was accurate. Moreover, the mean error (0.36) between the AI and manual labelling was smaller than that reported by Hwang et al. [13] (1.46 mm), which could be attributed to the fact that only cervical vertebral points were labelled in this present study while all cephalometric points were identified in the previous study (Hwang et al.). The precise point labelling ensured the accuracy and precision of linear measurements. Likewise, we found that the five linear and ratio measurements evaluating the morphology of cervical vertebrae through the AI algorithm were highly consistent with those through manual methods.

In this study, we developed a CNN model to label the contours of cervical vertebrae and to assess the CVM staging. As an optimal feature extractor applied at image positions, CNN is highly efficient for image processing [33,34]. The architecture of a typical CNN contains three layers: convolutional layers, pooling layers and fully connected layers. In convolutional layers, features of each image were extracted and organized in feature maps. These similar features were merged into new feature maps in pooling layers. Finally, all features were connected and classified in fully connected layers to output a predicted image [35].

Although six maturation stages are defined by the CVM staging according to different morphological shapes of cervical vertebrae, CVM stages are sometimes still difficult to differentiate since the morphological changes of cervical vertebrae are continuous rather than incremental. Thus, CS1 (no development) and CS6 (maturity) stages are easier to identify while the CS2–CS5 (in the process of development) stages are more difficult to differentiate. Consistently, our results revealed that AI performed best at CS1 (F1 score: 77%) and CS6 (F1 score: 85%).CS3 (growth spurt) showed the lowest F1 score (31%). Another explanation is the insufficient training set of CS3, for growth spurt is short and difficult to encounter in clinical practice. This finding was in accordance with that in Kok et al. [13]. Moreover, we found that the overall ICC between the AI staging and the gold standard was 0.98, indicating that the CNN model in this present study was accurate and precise in the assessment of CVM staging.

One of the limitations of our study was the size of testing dataset. In our study, we used 10% hold-out validation to test AI performance. This may result in over-fitting since the dataset was not properly distributed [36]. This may explain high precision and recall were detected for CS5 (recall: 100%) and CS6 (precision: 100%, specificity: 100%) stages. Therefore, we used F1 score to comprehensively evaluated AI performance.

Another limitation was the number of examiners in labelling. All the images were labelled by examiner 1. To keep labelling consistency, it was done twice after three months. The mean differences between the first and second manual labelling indicated a well consistency. Considering the labelling error, the midpoint of two manual labellings were saved as gold standard.

## 5. Conclusions

Taken together, we suggest that the AI algorithm described in this study is accurate and reliable in identifying the contours of cervical vertebrae and in CVM staging, with high accuracy (F1 score up to 85%) and perfect agreement with gold standard (ICC = 0.98).

## Figures and Tables

**Table 1:** The age, gender and CVM staging distribution of the dataset.

	Training	Testing
Age	12.02 ± 4.71	14.27 ± 4.78
Gender (M/F)	432/548	43/57
CS1	242	13
CS2	214	11
CS3	164	5
CS4	108	24
CS5	52	12
CS6	200	35
Total	980	100

**Table 2:** Landmarks and measurements used to determine cervical vertebral morphology.

Landmarks/ Measurements	Definition
C2lp	The most posterior point of C2 on the lower border
C2la	The most anterior point of C2 on the lower border
C2m	The deepest point of the concavity at the lower border of C2
C2Conc	The distance between C2m and the line connecting C2lp and C2la
C3up	The most posterior point of C3 on the upper border
C3ua	The most anterior point of C3 on the upper border
C3lp	The most posterior point of C3 on the lower border
C3la	The most anterior point of C3 on the lower border
C3m	The deepest point of the concavity at the lower border of C3
C3Conc	The distance between C3m and the line connecting C3lp and C3la
C3BAR	Ratio between the length of the base (distance C3lp − C3la) and the anterior height (distance C3ua − C3la) of the body of C3.
C4up	The most posterior point of C4 on the upper border
C4ua	The most posterior point of C4 on the upper border
C4lp	The most posterior point of C4 on the lower border
C4la	The most anterior point of C4 on the lower border
C4m	The deepest point of the concavity at the lower border of C4
C4Conc	The distance between C4m and the line connecting C4lp and C4la
C4BAR	ratio between the length of the base (distance C4lp − C4la) and the anterior height (distance C4ua − C4la) of the body of C4

**Table 3:** Landmarks and measurements used to determine cervical vertebral morphology.

Landmarks	Between Human Examiners		Between AI and Human Examiner	
	Mean ± SD (mm)		Mean ± SD (mm)	
C2lp	0.38	0.23	0.29	0.25
C2lm	0.70	0.44	0.47	0.33
C2la	0.41	0.28	0.35	0.27
C3up	0.56	0.40	0.48	0.38
C3ua	0.54	0.28	0.42	0.24
C3lp	0.41	0.24	0.24	0.15
C3m	0.57	0.37	0.37	0.28
C3la	0.32	0.19	0.24	0.17
C4up	0.50	0.29	0.42	0.36
C4ua	0.53	0.30	0.43	0.27
C4lp	0.37	0.21	0.25	0.21
C4m	0.62	0.45	0.46	0.27
C4la	0.35	0.20	0.27	0.18
Total	0.48	0.12	0.36	0.09

**Table 4:** The evaluation of CVM staging through AI.

	Precision/PPV	Recall/Sensitivity	Specificity	F1 Score
CS1	0.67	0.92	0.93	0.77
CS2	1.00	0.36	1.00	0.53
CS3	0.25	0.40	0.94	0.31
CS4	0.83	0.63	0.96	0.71
CS5	0.46	1.00	0.84	0.63
CS6	1.00	0.74	1.00	0.85
Accuracy	0.71			
ICC	0.98			

## Data Availability

Data sharing not applicable.
